# The pregnancy experiences and antenatal care services of Chinese migrants in Switzerland: a qualitative study

**DOI:** 10.1186/s12884-022-04444-1

**Published:** 2022-02-22

**Authors:** Dingcui Cai, Paulina Villanueva, Susannah Stuijfzand, Hong Lu, Basile Zimmermann, Antje Horsch

**Affiliations:** 1 Institute of Higher Education and Research in Healthcare (IUFRS), University of Lausanne, Route de la Corniche 10, Lausanne, 1011 Switzerland; 2 School of Nursing, Peking University, 38 Xueyuan Road, Haidian District, Beijing, 100191 China; 3 Confucius Institute, University of Geneva, Rue de Candolle, Geneva, 1211 Switzerland; 4 Department Woman-Mother-Child, Lausanne University Hospital and University of Lausanne, Lausanne, 1011 Switzerland

**Keywords:** Pregnancy experiences, Antenatal care services, Chinese, Migrant, Mothers, Fathers, Grandparents, Qualitative, Switzerland

## Abstract

**Background:**

Differences in reproductive health outcomes according to the mothers’ origins have been reported in Switzerland, for example, women from European countries and non-European countries. The Swiss Federal Office of Public Health has therefore called for specific Swiss-wide studies on migrant populations. This study explores the pregnancy and antenatal care experiences of Chinese migrants in Switzerland, intending to clarify their maternity care needs.

**Methods:**

In-depth interviews of 14 Chinese mothers and 13 family members were conducted in Chinese or English and audio recorded. All audio-recordings were transcribed verbatim. All Chinese transcripts were translated into English. Thematic analysis was performed with the assistance of the qualitative data analysis software, MAXQDA Analytics Pro 2020.

**Results:**

Five themes were extracted from the transcripts: (1) Motivations and concerns about having children, (2) The merits of the Swiss maternity care system, (3) The inconveniences and barriers of accessing Swiss maternity care services, (4) Strategies to deal with the inconveniences of the Swiss maternity care system, and (5) The need for culturally sensitive care.

**Conclusions:**

The results of our study provide new knowledge and understanding of pregnancy experiences and antenatal care services of Chinese mothers and their families in Switzerland. Their unique positive experiences included: family planning, the continuity of maternity services, humane care with the privacy respected, personalized sensitive care needs, preferences for female obstetricians and obstetricians of Asian origin. Several barriers were highlighted, such as information seeking difficulties, communication difficulties, and a rigid appointment system. Reducing barriers enabling access to maternity care services within the Swiss healthcare system is necessary to provide equal quality maternity care for individuals, irrespective of their origins.

## Background

According to the International Organization for Migration, approximately 272 million migrants^1^ worldwide live in a country other than their country of origin [1]. Europe hosts around 82 million migrants, comprising 30% of the global migrant population [1]. Switzerland, located on the European continent, has a large proportion of the foreign population. According to official statistics, 25.3% of the Swiss permanent resident population are migrants [2]. In the last few decades, the Swiss population growth was mainly attributable to net migration and, to a lesser extent, an excess of births over deaths [3]. Children born in Switzerland from foreign-born mothers account for 29.4% of births in Switzerland [2], compared to 20.9% in 27 countries of the European Union (excluding the UK) [4].

Differences in reproductive health outcomes according to the mothers’ origins have been reported in Switzerland [5]. Currently, reproductive health outcomes of women from European countries seem not to differ much from those of Swiss women [6], which might be because they often face few cultural differences^2^ and migration issues. For instance, they usually have a long-term residence permit in Switzerland and are well adapted to living in an international environment [8]. Comparatively, women from non-European countries seem often less able to realize their reproductive health potentials (i.e., good physical and psychological state of health during the pregnant and postpartum period) in Switzerland [9]. The issues leading to their difficulties of accessing healthcare and their poor reproductive health outcomes include migration-related reasons (e.g., discrimination, uncertain residence status, and poor language skills), poor social-economic status (e.g., low wages or unemployment), and inadequate support from the healthcare system (e.g., the burden of healthcare payments, insensitive care, insufficient health information and communication difficulties) [10–12].

To reduce maternal health disparities between different migrant sub-groups in Switzerland, the Federal Office of Public Health of Switzerland has called for specific Swiss-wide studies on migrant populations [13]. Asian minority groups from more than 17 Asian countries account for 10% of the total Swiss foreign population, where Chinese citizens (n = approximately 18,000) are the third-largest group of Asian migrants [14]. We expect some of our findings on the Chinese migrant group could facilitate the understanding of mothers of other Asian cultural groups and their childbirth experiences in Switzerland [15, 16]. They have so far not been systematically reported in the Swiss context. Therefore, in this study, we explored the experiences of pregnancy and maternity care services among Chinese migrants in Switzerland, with the goals of identifying their pregnancy outcomes and clarifying their maternity care needs.

## Methods

This qualitative study employed semi-structured in-depth interviews to explore the experiences of pregnancy and maternity care of Chinese migrants in Switzerland.

## Participants

Two groups of participants were recruited: (a) Chinese mothers and (b) family members including fathers (i.e., mothers’ partners) and grandparents (i.e., mothers’ parents or parents-in-law). In total, we aimed to recruit a minimum of 10 Chinese mothers and 10 family members. The sample size was based on similar published qualitative studies [17, 18]. In practice, new themes stopped emerging after 10 interviews. We ended up interviewing 14 Chinese mothers and 13 of their family members.

To be eligible for this study, mothers had to fulfill the following criteria: (a) were born and grew up in China and have Chinese nationality, (b) had a temporal or permanent Swiss resident permit, (c) remained in Switzerland during the data collection period, (d) have been pregnant or given birth within the previous one year, and (e) without significant pregnancy or postpartum health complications. It should be noted that the criteria did not exclude participants if they had temporally lived outside of China before their relocation to Switzerland, as our study focused on their unique childbirth experiences in the Swiss context. For family members, they had to remain in Switzerland during the data collection period, and they were not required to be Chinese born.

## Procedures

A Chinese social media platform, named WeChat, was used to recruit participants. The Ph.D. candidate (DC) in charge of data collection joined the WeChat groups of Chinese migrants in the French-, German- and Italian-speaking regions of Switzerland. Research flyers were posted in these WeChat groups to invite Chinese mothers and their family members to participate in the study. Eligible individuals who were interested in the study were contacted individually via WeChat to explain the nature of the study, the purpose, the procedures, and the expected duration. Once they agreed to participate in the study, an appointment was scheduled to conduct the interview. Prior to the interview, Chinese mothers and their family members were provided with an information sheet and a consent form in Chinese or English, depending on their request. They were given enough time to read the information and ask questions. After signing the consent form, the interview was conducted.

The semi-structured interviews with open-ended questions focused on their family planning, antenatal care, physical, and mental health during the pregnancy period. An opportunity was also given to all participants at the end of the interview to raise issues that they perceived to be important or that had not been covered. In addition, information on participants’ socio-demographic characteristics (e.g., age, marital status, education, occupation, residence duration, language skills, etc.) was collected.

All interviews were conducted by the Ph.D. candidate (DC), except for one interview, which was done by the research assistant (PV) due to a scheduling conflict. Most interviews (n=21) were conducted face-to-face individually at participants’ homes from November 2019 to February 2020. A few interviews (n=6) were carried out online between March and April 2020 due to the travel restriction of the Covid-19 lockdown in Switzerland. Interviews with Chinese and non-Chinese participants were conducted in Chinese and English, respectively, and audio recorded. All audio recordings were then transcribed verbatim. All Chinese transcripts were translated into English for data analyses by two Chinese postgraduate students with professional working proficiency in English and majoring in Nursing Science. English translations from Chinese and English quotations in the paper were validated by DC and BZ.

To protect participants’ privacy and maintain confidentiality on participant’s personal information, numbered names were used to represent mothers and their family members, respectively, in the data translation, analysis, and the paper writing (i.e., M = mothers; FM = family members). For example, “01-M” in the quote represents participant one of the mothers. The study was approved by the Commission cantonale d’éthique de la recherche CCER (project number: 2019-01734).

## Data analysis

Data analysis was performed following the step-by-step guidelines of Braun and Clarke [19]. Thematic analysis was employed to develop a rich thematic description and to analyze patterns of shared meanings of the experiences of the participants, using a qualitative data analysis software (MAXQDA Analytics Pro 2020).

Two analysts (DC and PV), one from Chinese background and the other from non-Chinese cultural background, coded the data separately in order to reduce personal biases. Before data analysis, the analysts discussed their preconceptions and expectations regarding the study results. After reading the verbatim transcripts several times to get an overall impression of the data, a provisional coding frame emerged. Based on this, the verbatim transcript of the first interview was coded separately by DC and PV to extract themes and sub-themes. The coding was then discussed and compared, and a consensus was reached to form the initial coding tree. The verbatim transcripts of the other interviews were analyzed using the same method. Results were discussed by the whole research team, and following their critical feedback, the final coding tree was obtained. No new codes emerged after the analysis of all the interviews.

## Results

During the data collection stage, there were 18 families who expressed interest in participating in the study. Eventually, 14 families agreed to do interviews. Participants were not screened further, as long as the eligibility criteria were met. A total of 27 participants from 14 families were recruited across Switzerland in eight States (Cantons): 14 Chinese mothers and 13 family members. The 13 family members included nine fathers (six Chinese and three non-Chinese fathers) and four Chinese grandparents (three maternal and one paternal grandparent). The three non-Chinese fathers were from Switzerland, Germany, or France.

All participants were officially documented and had legal residence permits. Almost all parents had a bachelor or higher degree, especially all fathers, who had master degrees or doctorates. There was no change in fathers’ employment status before and after moving to Switzerland. However, only five out of nine originally employed mothers maintained their employment status after moving to Switzerland, whereas four lost their jobs.

The number of years that parents had been living in Switzerland ranged from nine months to 21 years. Four grandparents lived in China before they paid a short visit to Switzerland. Detailed demographic information on mothers and fathers is shown in Table 1.

**Table 1:** Participants’ characteristics of mothers and fathers (n=23. Information for the four grandparents was not included in this table)

	Mothers (n=14)	Fathers (n=9)
	Mean (SD)/ N (%)	Mean (SD)/ N (%)
Mean age	34 (SD=4.27)	38 (SD=6.98)
< 30	2(14)	1(11)
30-35	8(57)	3(33)
36-40	3(21)	3(33)
> 40	1(7)	2(22)
Country of origin		
China	14 (100)	6 (67)
Country other than China	–	3 (33)
Marital status		
Married	13 (93)	8 (89)
Co-habiting	1 (7)	1 (11)
Mean number of years in Switzerland	5 (SD=5.26)	6 (SD=5.57)
≤ 2 years	5 (36)	3 (33)
3-6 years	5 (36)	2 (22)
7-10 years	3 (21)	2 (22)
≥ 11 years	1 (7)	2 (22)
Education		
Junior college/High School	1 (7)	–
Bachelor	4 (29)	–
Master	8 (57)	6 (67)
Doctor	1 (7)	3 (33)
Employment status		
Employed	5 (36)	9 (100)
Unemployed	9 (64)	–
Language skills		
French	6 (43)	5 (56)
German	2 (14)	2 (22)
English	12 (86)	9 (100)
Household income (USD/year)^3^	N (%) of family	
Below 60,000	3 (21)	
60,000 – 100,000	4 (29)	
100,000 – 200,000	6 (43)	
> 200,000	1 (7)	
Number of children	N (%) of family	
1	9 (64)	
2	4 (29)	
3	1 (7)	
Gender of the youngest child	N (%) of family	
Male	6 (43)	
Female	8 (57)	

The following five main themes emerged: (1) Motivations and concerns about having children, (2) The merits of the Swiss maternity care system, (3) The inconveniences and barriers of accessing Swiss maternity care services, (4) Strategies to deal with the inconveniences of accessing Swiss maternity care services, and (5) The need for culturally sensitive care. An overview of the main themes and sub-themes is shown in Table 2.

1. Motivations and concerns about having children
  1.1 Motivations for having children
    In this study, nine families had their first child, and five had more than one child. Chinese mothers and their family members reported that their motivations for raising children in Switzerland including good living conditions, filling the career gaps, reproductive stress from their extended families, as well as providing a companion and support from siblings.
    Chinese mothers and their family members reported their enjoyment of good living conditions in Switzerland. They favored the slow pace of work and life, convenient public facilities, as well as a peaceful environment with good food safety and social security, all of which encouraged them to have a baby in Switzerland. They also described having low parenting stress in the Swiss context.
    *“The good thing is that it is relatively calm here. It is suitable for giving birth in all aspects.” (05-FM)*
    Filling the career gaps was a common reason for Chinese mothers to have children in this study. This can be explained by the fact that most mothers in our study moved to Switzerland to accompany their Chinese husbands for work/study relocation or reunite with their foreign partners from cross-cultural marriages. It was challenging for those mothers to find a job because of migration issues and language problems. Thus, those families decided to have children during the gap years of the mothers’ careers.
    *“I came to Switzerland for my Ph.D. study, and my wife came with me. It is not realistic for her to find a job in a short time. Most families I’ve known in this case chose to have a baby first.” (01-FM)*
    Some mothers within “Chinese-Chinese” families (i.e., both parents were of Chinese origins) reported that, although they lived far away from their home country, they felt under great pressure to have a child from their extended families in China.
    *“Our family wanted us to have a baby very urgently. To be honest, we had been under great pressure from our parents.” (02-M)*
    The main motivation for families having more than one child in this study, particularly for mothers within “Chinese-foreign” families (i.e., Chinese wife and foreign husband), were for their children to have a companion and life-long support for their siblings.
    *“I was alone here with my husband. It was very hard. I had to rely on myself for everything. I was very tired every day. I just thought that our eldest son should have a sibling to support him in the future.” (12-M)*
  1.2 Concerns about having further children
    Although five families had a second or third child in our study, the other families with one child expressed their negative concerns about having further children. In general, the lack of family support regarding childcare was a common concern. This was notable among the “Chinese-Chinese” families, due to international travel restrictions and short-term visitors’ visas for Chinese family members visiting Switzerland.
    *“Because our family is a ‘Chinese-Chinese’ family, we have no friends and relatives here to help us. I have no plan to have a second child.” (07-M)*
    Mothers within “Chinese-foreign” families reported that the support from their foreign partners’ extended families was also very limited. They described that due to cultural differences, their foreign parents-in-law from Swiss and European cultural backgrounds (Switzerland, Germany, or France) were less likely to be involved in childcare than their Chinese parents. Thus, two mothers in our study had to send their oldest child to China to be cared for by their Chinese parents after they had the second child.
    *“I sent my oldest son back to China and let my mom take care of him. Fortunately, my mom can help me, otherwise, I would be exhausted.” (13-M)*
    In addition, balancing between career development as migrants and the parenting roles had a major impact on some families regarding their decisions on whether to have further children or not. The five (out of 14) mothers in this study who were employed expressed their concerns about the interruption of their careers due to childbearing responsibilities while they struggled to settle down as migrants in Switzerland. Consequently, those families were heavily inclined to not have a second child.
    *“We completed our Ph.D. here. Because we want to settle down here and we must develop our careers. It’s impossible for my wife to stay at home to only take care of children.” (13-FM)*
    Finally, parents within “Chinese-Chinese” families described how the “One-Child” policy in China had affected their family planning. Some parents reported that, by being the only child in their extended family, they would be under great pressure to support four elderly parents and raise at least one child at the same time when they return to China shortly. Therefore, they were inclined not to have a second child.
    *“I think the ‘One-Child’ policy has put much stress on our generation. [ …] Because my husband and I are the only children in our extended family. If we have two children, then we will be under great pressure of raising two children and taking care of four elderly parents at the same time.” (04-M)*
    Chinese-Chinese families with only female children expressed not being concerned by the “son preference” Chinese tradition. Some parents expressed that it was best for a family to have a gender balance of children, and some shared a slight inclination to have daughters. They believed that daughters are more reliable than sons to provide emotional support for parents during their old age.

2. The merits of the Swiss healthcare system
  In general, Chinese mothers and their families perceived that maternity care services in Switzerland were better than those in their home country. The continuity of the maternity services, the comprehensive coverage of health insurance, and the conveniences of the appointment system all reported contributing to their positive maternity care experiences with the Swiss healthcare system. Additionally, humane care with the respect of privacy was highly valued by Chinese mothers in this study.
  2.1 The continuity of the maternity care services
    Usually, Chinese mothers had a private obstetrician responsible for their antenatal care in this study. This was perceived as one of the great benefits for Chinese mothers of being pregnant in Switzerland. With a private obstetrician, Chinese mothers appreciated the advantage of being followed up by the same obstetrician during the pregnancy period.
    *“Every mother here has her private obstetrician. The obstetrician has all the information of the mother, so he/she understands the mother’s situation very well.” (13-M)*
  2.2 The comprehensive coverage of health insurance
    The comprehensive coverage of health insurance was reported to be better than Chinese mothers’ expectations. Chinese mothers explained that they only needed to pay the basic maternity insurance, and their maternity services could be fully covered from pregnancy to postpartum home visits. Therefore, they did not worry about childbirth costs in Switzerland.
    *“Most mothers choose to give birth here because they don’t need to worry about the cost. All costs related to childbirth are covered by the insurance. You could simply follow advises that doctors give you without worrying about cost.” (01-FM)*
  2.3 The conveniences of the appointment system
    Parents expressed their appreciation for the appointment system of their maternity care services in Switzerland. Parents explained that being able to go to hospitals at the designated appointment time helped them to reduce the unnecessary waiting time. Furthermore, the maternity care providers were only responsible for one patient during the appointment time. They were allocated sufficient time for in-depth consultations related to their pregnancy-related health concerns.
    *“It is all one-to-one service here when you visit your doctor. Also, there is no need to wait in line when we go to the hospital. Unlike in China, the hospitals are overcrowded, and you need to wait a long time to register and see a doctor.” (13-FM)*
  2.4 Humane care with privacy respected
    Chinese mothers and their family members considered their interactions with their maternity care providers as highly positive. They positively commented on trustworthy, humane, and friendly services from their maternity care providers, which made them feel secure and relaxed in a new country. In addition, they expressed their great satisfaction with the way mothers’ privacy was protected during their consultation visits and obstetric examinations.
    *“I’ve been in hospitals in China, and privacy is not particularly a concern in Chinese hospitals, while we have it in Switzerland.” (09-FM)*

3 The inconveniences and barriers of accessing Swiss maternity care services
  As foreigners being pregnant in a foreign country, mothers and their families appreciated the merits of Swiss maternity care services. Nevertheless, they faced challenges. The inconveniences and difficulties for Chinese mothers accessing maternity care services in Switzerland were mainly found to originate from the language barrier and the rigid appointment system.
  3.1 The language barrier
    The language barrier was the most significant challenge for most Chinese mothers and their families accessing maternity care services in this study. It was related to two aspects: one was an obstacle to information seeking, and the other was related to communication difficulties.
    In terms of information seeking, some mothers in this study expressed that they were not aware that German and French rather than English are the main languages spoken in Switzerland. They were not linguistically prepared before coming to Switzerland. Seven of 14 Chinese mothers who could not speak any local language reported that they gave up the opportunity to participate in prenatal courses because of language limitations. In addition, they could not understand written information distributed by hospitals.
    *“They gave me a lot of paper information, but they were all in German. I asked them whether they had an English version, but they said no. After taking them back, I read them with Google Translate, but I lost interest and patience pretty quickly (laughs). I then put them aside.”(08-M)*
    Two mothers asked a private midwife to give them one-to-one prenatal courses because they could not communicate with the attending midwife and other mothers in the local language. They regretted the missed opportunities of exchanging information with other mothers and the midwife in group classes.
    *“The private midwife gave us a one-on-one prenatal class in English that lasted about 1.5 hours. However, the more important role of the prenatal class was for the participating families to share their experiences. Because we were in a private one-on-one session, we lost the opportunity to interact with other families.” (08-M)*
    Chinese mothers with local language limitations reported that it was not always guaranteed that they would be able to effectively communicate with their maternity care providers in English without misunderstanding, especially in cases when their maternity care workers did not speak English well.
    *“Because we are not native English speakers, even if the doctor’s English is excellent, we couldn’t understand him in many aspects. [ ⋯] Particularly when we speak English to some nurses, they reply to us in German or the simplest English with some body language.” (08-M)*
    Some of those mothers reported that they could not discuss and ask their maternity care providers for detailed information due to the language barrier. Some mothers expressed that they felt hesitant to ask questions because of poor language competency and a lack of courage as foreigners living in Switzerland.
    *“We live here as foreigners. We don’t have the courage or language ability to discuss health issues with doctors. [ ⋯] Most of the time, it’s embarrassing for me to ask doctors questions.” (05-M)*
    It is noteworthy that for those mothers who had local language skills, this did not necessarily mean that they could easily have smooth and effective discussions with their maternity care providers. Some of them often found that they did not understand medical terminologies.
    *“We have been living in France for 10 years before moving to Switzerland. There is no big language barrier for me in French communication in daily life, but I still didn’t understand medical terminologies when I visited my obstetrician and gave birth at the hospital.” (02-M)*
    An additional and unique challenge regarding the communication difficulties was that four official languages are spoken in different regions across Switzerland. Two mothers moved from one language zone to another due to their husbands’ job relocation, after they had put much effort and energy to master one local language. They faced communication difficulties in adapting to yet another language in the new language zone.
    *“In Geneva, my French was not a big problem. [ ⋯] However, because I could not speak German, there were communication problems in Zurich hospitals when we moved there due to my husband’s job change.” (10-M)*
  3.2 The rigid appointment system
    Another inconvenience reported was related to the organization of the appointment system to visit hospitals. Chinese mothers expressed that the Swiss healthcare system was quite different from that in their home country. An appointment in advance was always required for every hospital visit, whereas there were possibilities of walk-in consultations in Chinese hospitals. Although scheduling appointments via phone calls saved time for registration in Swiss hospitals, the waiting time for the appointment was often long. Some mothers reported being anxious during the long waiting time. Some others were worried that they had missed out on some of their routine visits to their doctors.
    *“I was very anxious throughout my pregnancy. Why did other mothers have a monthly maternity checkup, while I had to wait for a month and a half? It was my first pregnancy. I didn’t know anything. During the waiting time, I was very worried and anxious.” (07-M)*

4 Strategies to deal with the inconveniences of accessing Swiss maternity care services
  To overcome the inconveniences of accessing Swiss maternity care services, Chinese mothers in this study explored alternative ways to obtain information and assistance in communication with their maternity care providers.
  4.1 Strategies ways of information seeking
    Chinese mothers reported that they turned to websites, mobile applications, and books in Chinese when they could not gain essential knowledge and information on pregnancy and childbirth from their maternity care providers. In addition, some mothers shared that it was beneficial for them to exchange pregnancy experiences with other Chinese mothers and obtain information guidelines on their personalized needs. The knowledge and support they received through these channels were perceived to meet their information needs and largely addressed their concerns.
    *“Mainly from Chinese websites. Some Chinese mothers also told me their experiences and knowledge. Based on the information they told me, and then I searched online and checked it by myself.” (06-M)*
    However, the reliance on the native language for information seeking sometimes further discouraged Chinese mothers from seeking essential local medical information. Some mothers in this study indeed reported that they could not understand the healthcare system and medical procedures within the Swiss healthcare system.
    *“We didn’t understand healthcare system and medical procedures here.” (07-M)*
  4.2 Communication assistance
    To overcome communication difficulties, some mothers used translation services, with mixed outcomes. One mother reported that she appreciated the free-of-charge Chinese translation services for prenatal courses and important consultations with her obstetrician at a French-speaking university hospital. However, another mother shared that she did not use the translation services at a German-speaking university hospital. She explained that the multi-lingual midwife who served as the translator could not correctly understand her.
    *“We didn’t use it. It’s not a professional translation service. They just found a midwife with multiple language skills responsible for taking care of the mothers who speak languages like French, German, English, Spanish” (05-M)*
    Alternative measures included introducing assistance from women’s husbands with accessing maternity care services and communicating with maternity care providers. This was often largely due to the more advanced English or local language skills of women’s husbands. Fathers in this study also confirmed that when there was no professional assistance, they were the ones who could be relied on for language assistance.
    *“When my wife was pregnant, I was basically in charge of all things, such as booking appointments and communicating with the doctors.” (01-FM)*

5 The need for culturally sensitive care
  The culturally sensitive care needs of Chinese mothers were mainly related to three aspects in this study: personalized sensitive care, preferences for female obstetricians, and obstetricians of Asian origin.
  5.1 Personalized sensitive care
    Some Chinese mothers in our study reported that sometimes their views conflict with those of their obstetricians due to what they perceived as cultural differences. Chinese mothers believed that some precautionary treatments were necessary when they suffered certain pregnancy symptoms, such as prolonged vaginal bleeding, placenta previa, and advanced pregnancies. However their obstetricians expressed that pregnancy symptoms are natural and were not a sign of sickness. Therefore, there was no need for special attentive care. As a result, mothers considered that their pregnancy-related issues failed to raise the attention of their obstetricians, which caused a sense of insecurity or anxiety during their pregnancy period.
    *“When I was being pregnant for four or five months, I still had vaginal bleeding. I called my obstetrician and he said that I didn’t need to pay too much attention and I could live a normal life. I searched the information on the Internet. It said that in my case, it was necessary to do some precautionary treatments. Otherwise, it was easy to cause miscarriage. I felt that I needed to see my obstetrician, but he said it was unnecessary. I was very worried and anxious during that time.” (13-M)*
  5.2 Personalized for female obstetricians
    Some Chinese mothers described preferring female obstetricians in our study. They explained that childbirth was a woman’s private matter and that they did not feel ashamed and embarrassed to expose their bodies in front of female obstetricians. In addition, they perceived that female obstetricians who had childbearing experiences were more empathetic to their maternity care needs than their male colleagues.
    *“It seems like it doesn’t matter for people here to have a male obstetrician. [ ⋯] However, I felt more natural to find a female obstetrician when I was pregnant for my third child. Therefore, I changed my male doctor (to a female one).” (11-M)*
  5.3 Obstetricians of Asian origin
    One mother chose an obstetrician of Asian origin, as she believed that the obstetrician from a similar cultural background would be more sensitive to her needs, such as emotional support and sensitivity to her pregnancy symptoms.
    *“My friend recommended me a private obstetrician. He is Vietnamese. He was very patient with my complaints. He comforted me a lot during my pregnancy.” (02-M)*

**Table 2:** Main themes and sub-themes of this study

Main themes	Sub-themes
1. Motivations and concerns about having children	1.1 Motivations for having children
	1.2 Concerns about having further children
2. The merits of the Swiss maternity care system	2.1 The continuity of the maternity care services
	2.2 The comprehensive coverage of health insurance
	2.3 The conveniences of the appointment system
	2.4 Humane care with privacy respected
3. The inconveniences and barriers of accessing Swiss maternity care services	3.1 The language barrier
	3.2 The rigid appointment system
4. Strategies to deal with the inconveniences of the Swiss maternity care system	4.1 Alternative ways of information seeking
	4.2 Communication assistance
5. The need for culturally sensitive care	5.1 Personalized sensitive care
	5.2 Preferences for female obstetricians
	5.3 Obstetricians of Asian origin

## Discussions

To our knowledge, this is the first qualitative study exploring the experiences of pregnancy and maternity care services of Chinese mothers in Switzerland. We found five main themes, namely (1) motivations and concerns about having children, (2) the merits of the Swiss maternity care system, (3) the inconveniences and barriers of accessing Swiss maternity care services, (4) strategies to deal with the inconveniences of accessing Swiss maternity care services, and (5) the need for culturally sensitive care.

In our study, the Chinese mothers in Switzerland generally had positive pregnancy experiences and were satisfied with the maternity care services they received. A highly appreciated quality of the Swiss healthcare system was the continuity of maternity care services provided by the same obstetricians throughout their pregnancies. Their satisfactions partially came from comparing their previous medical experiences in their home country, where the doctors for consultations are not fixed. In line with the previous study [20], our study confirmed that the follow-up by the same obstetrician allows the establishment of trusting relationships between pregnant mothers and their healthcare providers. In addition, it helps the avoidance of retelling or relearning mothers’ medical history for each consultation [21]. While these mutual benefits are understandable in general, our study revealed and emphasized the importance of such continuity of services for Chinese mothers in the unique Swiss context. Chinese mothers living in Switzerland have a language disadvantage. Thus, they rely on having the same obstetrician to minimize misunderstandings in their communication. Additionally, it improves Chinese mothers’ mental health during their pregnancies by creating a relaxed and secure healthcare environment. It would be natural to expect this finding could be extended to the understanding and the optimization of the maternity care services for other minority groups in a foreign country.

Chinese mothers in our study praised the one-to-one consultations with their obstetricians and described the maternity care services they received as humane, patient, and friendly. They appreciated their privacy being respected during their antenatal visits within the Swiss healthcare system. This principle is usually not emphasized in their home country, as it is not considered an essential ethical principle in Chinese hospitals [22]. Our finding corroborates global studies [23, 24] on the importance of maintaining the privacy and dignity of mothers throughout their maternity care due to the private nature of obstetrical examinations and medical procedures.

Our study found that the comprehensive coverage by the Swiss maternity insurances had well exceeded participating Chinese mothers’ expectations. This overwhelming approval could be understood considering their comparative experiences with medical care in China. While Swiss maternity insurance covers prenatal check-ups, prenatal classes, hospital delivery costs, and postnatal midwife visit services, comprehensive coverage is not universal in China. Maternity care insurances are only supplemental and usually obtained as employment benefits in China [25, 26].

Chinese mothers in our study had mixed feelings about the scheduling of their medical appointments within the Swiss healthcare system. On the positive side, the appointment service saved the lengthy waiting time whilst queuing at hospitals, compared with their medical experiences in China [27, 28]. On the negative side, some reported that they felt worried and anxious during the period between scheduling and the actual appointment. Due to the limits on the scope of our study, it is not yet clear whether the long latency for Chinese mothers’ antenatal appointments was due to the unavailability of language-competent maternity care providers or simply due to a mismatch of expectations between the Chinese mothers and their Swiss healthcare providers on the needed frequency for antenatal visits. A follow-up study on the actual causes might be needed in the future. Nevertheless, our study revealed the necessity to better inform Chinese mothers about the Swiss healthcare system and its functioning, in particular the procedures for proper medical attention. Another concern expressed by Chinese mothers regarding their antenatal appointments was the lack of walk-in consultations to get quick responses to their pregnancy-related worries. To address this concern, it would be beneficial for the obstetricians to assist and educate the Chinese mothers to determine when emergency interventions are needed and means of access throughout their pregnancies. It would be particularly useful for such information to be given during their first consultation. This would allow Chinese mothers to properly adjust their expectations and be better prepared, should there be a need to seek additional help for their potential pregnancy-related symptoms.

Our study found that not speaking the local language created the most obvious barrier for Chinese mothers accessing maternity care services in Switzerland. Nearly half of the Chinese mothers and their husbands in our study could not speak any local language. While it appears apparent that the language barrier would be normal among immigrants in a foreign country, the origins and features within the Swiss context are unique and believed to be overlooked. First of all, English is the dominating second language taught within the Chinese educational system. It is challenging for Chinese mothers to master a third language completely different from their mother tongue system, especially for those mothers who relocated to Switzerland during their pregnancies. Secondly, it has to be taken into consideration that Switzerland is a culturally diverse country with four national languages other than English (German, French, Italian, and Romansh) spoken across different regions [2]. The lack of a unified language poses another layer of difficulty for Chinese mothers when they move across language zones. Thirdly, our study revealed a clear gap in the mutual understanding between Chinese mothers and their healthcare providers on the grounds of language. Swiss healthcare providers were often not prepared to care for migrants who did not speak their local languages. On the other hand, some Chinese mothers in our study were found to have very limited knowledge of Switzerland before they arrived, including its diverse language system, and were thus insufficiently prepared for their new lives with a growing family.

It should be noted that the uniqueness of language barriers for Chinese mothers to access maternity care in Switzerland is very different from those Chinese mothers living in English-speaking developed countries, such as the US and Canada, which host large Chinese populations and where the availability of maternity care workers with desired language (Chinese) competences is much more feasible [21, 29]. This implies both complexity and difficulties for the delivery of quality maternity care services to Chinese migrants living in Switzerland. We observed several positive attempts that some Chinese mothers developed to overcome the inconveniences and reduce language barriers, including seeking information from the internet or mobile applications in the Chinese language, using interpreter services provided by the hospitals, and turning to their husbands with better language competencies. However, all these measures have their limitations, and none of them could completely address the challenges, given that maternity care is highly professional and personalized.

We believe great efforts remain to be made by both the Swiss healthcare system and individual migrants. It is essential to raise the awareness of such a language issue within the system and among its healthcare workers [11]. In addition, there are possibilities to improve the communication between mothers and healthcare workers with the assistance of new technologies, such as AI-powered real-time translations. The promotion of delivering healthcare-related information and services in English [12], as the only international language learned by the majority of the migrants, in paper-written forms, websites, and inpatient visits, is deemed highly effective to safeguard the core health-related interests of individual mothers. Despite language difficulties, Chinese mothers should still be encouraged to proactively engage with their maternity care workers in person, so that their reproductive health outcomes will not be comprised. Finally, the Swiss healthcare system had piloted the telephone interpreting services for emergencies and brief clarifications [30]. Further studies may propose to evaluate the possibilities of introducing such a service for non-emergency healthcare such as maternity care for migrant women.

It is noteworthy that despite Chinese mothers and Chinese migrant families in Switzerland were living far away from their home country, our study highlighted that Chinese cultural and social-economic developments remain to have certain influences on their family planning as well as the demands for culturally-specific care services. Their motivations for having a child were driven by family pressure, which is common in China [31]. Interestingly, although Swiss society provides a flexible and supportive environment for raising children, the consequences of the “One-Child” (ended in 2015) policy still have a major influence on how the Chinese mothers and their families feel about having further children. Mostly because of the economic pressure and psychological concerns over simultaneously raising multiple children and caring for elderly parents. In addition, the impact of cultural differences on the antenatal care experiences of Chinese mothers in Switzerland is evident. This is typically reflected in the conflicts of ideas and beliefs on the pregnancy process from Chinese mothers and their Swiss healthcare providers. The Chinese mothers desire culturally sensitive care instructions to adjust their lifestyles throughout their pregnancies [32] and also deal with pregnancy-related symptoms. Since the number of Chinese migrants in Switzerland remains small, unlike the situation reported by a study of Chinese women in Canada [21], we doubt the Swiss healthcare system could employ sufficient obstetricians with Chinese backgrounds to address such needs. Nevertheless, Swiss maternity care workers with different backgrounds than those Chinese mothers should encourage Chinese mothers to express their culture-specific concerns for better reproductive health outcomes.

## Strengths and limitations

This study has several strengths. The qualitative approach allowed mothers and their families to discuss their personal experiences and perceptions in-depth. The inclusion of family members such as husbands and mothers’ parents or parents-in-law contributed to the richness of the results. Furthermore, Chinese participants were interviewed in their native language Chinese, and foreign fathers in English, enabling them to tell their stories without the hindrances caused by language barriers. Regarding the data analysis, the data were analyzed by two researchers (DC and PV) with different cultural backgrounds (i.e., a Chinese migrant from an insider perspective and a Swiss citizen from an outsider perspective) to minimize any personal biases.

The study is limited by the lack of socio-demographic diversity in the participants. All participants were officially documented with legal residence permits. Moreover, participants tended to be highly educated, coming from higher social classes, and more approval motivated. Future research with attempts to include those more marginalized groups, such as undocumented Chinese migrants or those from lower socio-economic background, is needed. Finally, we acknowledge the voluntary participation could be another potential limitation in our study.

## Implications

The results of our study point to some ideas that may improve the Swiss maternity care services, if the following strategies could be implemented. (1) The language barrier was the most significant challenge for Chinese mothers in our study. Health information and services should be provided in English in written materials, on websites, during prenatal visits, as well as prenatal courses. Other communication channels for migrant mothers when accessing maternity care services should also be considered. Such as interpreter services, AI-powered real-time translations, and telephone interpreting services. (2) Although Chinese mothers in our study expressed satisfaction with the appointment system for their maternity care services, they also reported having pregnancy-related worries in-between their appointments. It would be beneficial to provide migrant mothers with detailed guidelines and explanations about precautionary treatments and accessing these services throughout their pregnancy period. (3) Chinese mothers in our study expressed they could not understand the healthcare system and medical procedures. Maternity care providers and health institutions should increase awareness that the common problem for migrant mothers is the lack of knowledge about what services within the Swiss healthcare system are available. Efforts are needed to help migrant mothers to familiarize themselves with the Swiss maternity care system and its services. (4) Overall, Chinese mothers in our study were satisfied with the maternity services within the Swiss healthcare system they received. At the same time, they expressed culturally sensitive care needs. Being culturally sensitive is essential for Swiss maternity care providers and adequate training should thus be provided. Our findings may help to understand the maternity care needs of Chinese mothers in other non-English speaking Western countries, which needs to be investigated in future studies.

## Conclusions

The results of our study provide new knowledge and understanding of pregnancy experiences and antenatal care services of Chinese migrants in Switzerland. Their unique experiences included: family planning, the continuity of maternity care, humane care with the privacy respected, personalized sensitive care needs, preferences for female obstetricians and obstetricians of Asian origin. Several barriers were highlighted, such as information seeking difficulties, communication difficulties, and a rigid appointment system. Reducing barriers enabling access to maternity care services within the Swiss healthcare system is necessary to provide equal quality maternity care for individuals, irrespective of their origins.

## Data Availability

Due to concerns of potential violations to the participants’ privacy, the individual data set generated during the present study is not publicly available. However, the final data set for data analysis is available from the corresponding author on reasonable request.

## Footnotes

1 The terms “migration” and “migrants” have been much debated among scholars. In this study, we focused on Chinese people whom we considered as individuals moving from one country to another, which may be considerably different from the one they are used to.

2 In this paper, we will use the term “culture” sparingly to refer to the broad idea of ways of living and thinking shared by a group of people (for an overview of the debates in anthropology on the concept of culture, see Kuper 1999 [7]).

3 Switzerland Annual Household Income per Capita reached 57,361.582 USD in 2018.
